# (*E*)-1-Ferrocenyl-3-(2-meth­oxy­phen­yl)prop-2-en-1-one

**DOI:** 10.1107/S1600536814003912

**Published:** 2014-02-26

**Authors:** Myrna R. Otaño Vega, Kennett I. Rivero, Ingrid Montes González

**Affiliations:** a Department of Chemistry, University of Puerto Rico, Río Piedras Campus, San Juan, Puerto Rico

## Abstract

The structure of the title compound, [Fe(C_5_H_5_)(C_15_H_13_O_2_)], consists of a ferrocenyl moiety and a 2-meth­oxy­phenyl group linked through a prop-2-en-1-one spacer in an *E* conformation. In the ferrocene unit, the substituted cyclo­penta­dienyl (Cps) ring and the unsubstituted cyclo­penta­dienyl ring (Cp) are almost parallel to one another [dihedral angle = 1.78 (14)°], and the Cp and Cps rings are in a *gauche* conformation. The benzene ring is twisted by 10.02 (14) and 11.38 (11)° with respect to the Cp and Cps rings, respectively. In the crystal, mol­ecules are linked by weak C—H⋯O hydrogen bonds into supra­molecular chains running along the *b*-axis direction.

## Related literature   

For the synthesis, see: Attar *et al.* (2011 ▶); Kumar *et al.* (2012 ▶). For related syntheses and background, see: Liu *et al.* (2001 ▶); Wu *et al.* (2002 ▶); Ji *et al.* (2003 ▶); Maree *et al.* (2008 ▶); Jiao *et al.* (2009 ▶); Cardona *et al.* (2010 ▶). For the biological activity of calcones and chalcone derivatives, see: Wu *et al.* (2002 ▶); Arezki *et al.* (2009 ▶); Nabi & Liu (2011 ▶); Zhao & Liu (2012 ▶). For related structures, see: Lindeman *et al.* (1997 ▶); Wu *et al.* (2006 ▶); Liu *et al.* (2008 ▶).
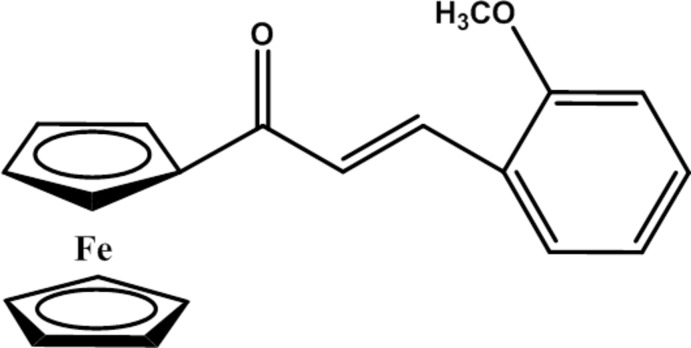


## Experimental   

### 

#### Crystal data  

[Fe(C_5_H_5_)(C_15_H_13_O_2_)]
*M* *_r_* = 346.19
Orthorhombic, 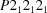

*a* = 8.8352 (1) Å
*b* = 11.4047 (1) Å
*c* = 16.1327 (2) Å
*V* = 1625.58 (3) Å^3^
*Z* = 4
Mo *K*α radiation
μ = 0.93 mm^−1^
*T* = 296 K
0.22 × 0.17 × 0.12 mm

#### Data collection  

Bruker APEXII CCD diffractometer
Absorption correction: multi-scan (*SADABS*; Bruker, 2007 ▶) *T*_min_ = 0.821, *T*_max_ = 0.896
13238 measured reflections
3659 independent reflections
3242 reflections with *I* > 2σ(*I*)
*R*_int_ = 0.022

#### Refinement  

*R*[*F* ^2^ > 2σ(*F* ^2^)] = 0.026
*wR*(*F* ^2^) = 0.065
*S* = 1.05
3658 reflections
209 parameters
H-atom parameters constrained
Δρ_max_ = 0.21 e Å^−3^
Δρ_min_ = −0.13 e Å^−3^
Absolute structure: Flack (1983 ▶), 1523 Friedel pairs
Absolute structure parameter: 0.004 (14)

### 

Data collection: *APEX2* (Bruker, 2007 ▶); cell refinement: *SAINT* (Bruker, 2007 ▶); data reduction: *SAINT*; program(s) used to solve structure: *SHELXS97* (Sheldrick, 2008 ▶); program(s) used to refine structure: *SHELXL97* (Sheldrick, 2008 ▶); molecular graphics: *SHELXTL* (Sheldrick, 2008 ▶); software used to prepare material for publication: *SHELXTL*.

## Supplementary Material

Crystal structure: contains datablock(s) I, New_Global_Publ_Block. DOI: 10.1107/S1600536814003912/xu5769sup1.cif

Structure factors: contains datablock(s) I. DOI: 10.1107/S1600536814003912/xu5769Isup2.hkl

CCDC reference: 987910

Additional supporting information:  crystallographic information; 3D view; checkCIF report

## Figures and Tables

**Table 1:** Hydrogen-bond geometry (Å, °)

*D*—H⋯*A*	*D*—H	H⋯*A*	*D*⋯*A*	*D*—H⋯*A*
C6—H6⋯O1^i^	0.93	2.48	3.368 (2)	159

Symmetry code: (i) 
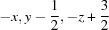
.

**Fig. 1.**
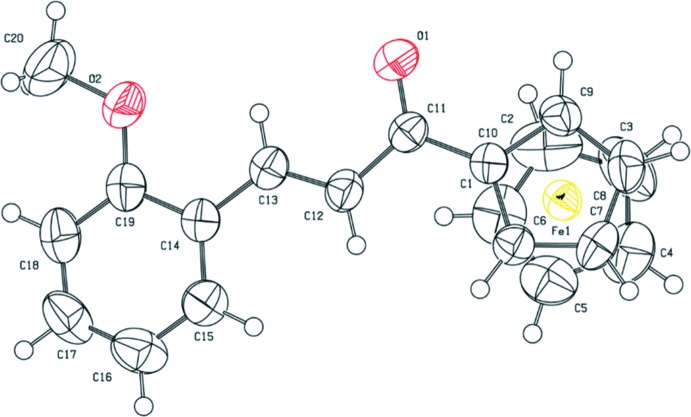
The molecular structure of the title compound.

## supplementary crystallographic information

### 1. Comment

Chalcones occur in nature as precursors of flavonoids and exhibit various biological activities such as anti-cancer, anti-inflammatory, nitric oxide regulation and anti-hyperglycemic agents (Liu *et al.*, 2001). They are traditionally synthesized in the laboratory, *via* the Claisen–Schmidt condensation carried out in basic or acidic media under homogeneous conditions (Attar *et al.*, 2011). Structural modifications of the chalcone template are readily achieved. Biological activities of chalcones are equally wide ranging, such as: anti-bacterial and anti-hyperglycemic, anti-malarial, anti-HIV, anti-oxidant, and anti-tumor (Wu *et al.*, 2002).

The crystal structure of the title compound reveals that the configuration about the C12═C13 bond corresponds to the (E)-isomer. The majority of the C and O atoms of the substituted cyclopentadienyl ring (Cps) are *sp*^2^-hybridized and the conjugation is lost at the methoxy substituent of C19. In the ferrocenyl moiety, the planes formed by the Cp (unsubstituted cyclopentadienyl ring) and Cps are almost parallel. The C atoms in these two rings have adopted a *gauche* conformation, and the Fe metal center lies closer to the Cps ring. The Fe—*Cg* and Fe—Cgs distances are 1.658 (2) and 1.644 (2) Å, respectively, where *Cg* and Cgs are the centroids of Cp and Cps, respectively. The *Cg*—Fe—Cgs angle is 178.4 (2)°.

### 2. Experimental

The title compound was synthesized according to the literature procedure (Cardona *et al.*, 2010). An aqueous solution of sodium hydroxide (5%, 2 ml) was added slowly with stirring to acetylferrocene (0.456 g, 0.002 mol). Then, 2-methoxybenzaldehyde (0.272 g, 0.002 mol) in ethanol (2 ml). The resulting mixture was stirred at room temperature for 2 h. The dark-orange-red precipitated solid was filtered off, washed with cold water and ethanol, dried and recrystallized from a mixture of acetone:water (yield, 84%; M·P. 144–145 °C). Dark violet crystals, suitable for X-ray diffraction, were obtained by the slow evaporation of a 1:1 (*v*/*v*) acetone:water solution of the title compound at room temperature over a period of 1 day. NMR analyses were performed on a Bruker AV-500 spectrometer by using CDCl_3_ 99.9% pure as a solvent and Me_4_Si as external standard.^1^H-NMR (δ in p.p.m., CDCl_3_): 3.90 (3*H*, s), 4.20 (5*H*, s), 4.60 (2*H*, s), 4.90 (2*H*, s), 7.05 (1*H*, d), 6.95, 7.25, 7.35, 8.10 (4*H*, dd, d,d, dd), 7.65 (1*H*, d). ^13^C-NMR (δ in p.p.m., CDCl_3_): 55.5, 69.7, 70.1, 72.5, 80.9, 111.2, 123.9, 120.7, 124.7, 128.9, 131.2, 136.3, 158.7, 193.5.

### 3. Refinement

H atoms were placed in calculated positions with C—H = 0.93–0.96 Å and refined in riding mode with U_iso_(H) = 1.5U_eq_(C) for methyl H atoms and 1.2U_eq_(C) for the others.

### Crystal data

**Table:**

[Fe(C_5_H_5_)(C_15_H_13_O_2_)]	*D*_x_ = 1.415 Mg m^−^^3^
*M**_r_* = 346.19	Melting point: 417 K
Orthorhombic, *P*2_1_2_1_2_1_	Mo *K*α radiation, λ = 0.71073 Å
Hall symbol: P 2ac 2ab	Cell parameters from 6681 reflections
*a* = 8.8352 (1) Å	θ = 2.9–26.8°
*b* = 11.4047 (1) Å	µ = 0.93 mm^−^^1^
*c* = 16.1327 (2) Å	*T* = 296 K
*V* = 1625.58 (3) Å^3^	Prism, red
*Z* = 4	0.22 × 0.17 × 0.12 mm
*F*(000) = 720	

### Data collection

**Table:**

Bruker APEXII CCD diffractometer	3659 independent reflections
Radiation source: fine-focus sealed tube	3242 reflections with *I* > 2σ(*I*)
Graphite monochromator	*R*_int_ = 0.022
φ and ω scans	θ_max_ = 27.5°, θ_min_ = 2.2°
Absorption correction: multi-scan (*SADABS*; Bruker, 2007)	*h* = −10→11
*T*_min_ = 0.821, *T*_max_ = 0.896	*k* = −14→14
13238 measured reflections	*l* = −20→20

### Refinement

**Table:**

Refinement on *F*^2^	Secondary atom site location: difference Fourier map
Least-squares matrix: full	Hydrogen site location: inferred from neighbouring sites
*R*[*F*^2^ > 2σ(*F*^2^)] = 0.026	H-atom parameters constrained
*wR*(*F*^2^) = 0.065	*w* = 1/[σ^2^(*F*_o_^2^) + (0.0341*P*)^2^ + 0.0721*P*] where *P* = (*F*_o_^2^ + 2*F*_c_^2^)/3
*S* = 1.05	(Δ/σ)_max_ = 0.002
3658 reflections	Δρ_max_ = 0.21 e Å^−^^3^
209 parameters	Δρ_min_ = −0.13 e Å^−^^3^
0 restraints	Absolute structure: Flack (1983), 1523 Friedel pairs
Primary atom site location: structure-invariant direct methods	Absolute structure parameter: 0.004 (14)

### Special details

**Table:**

Geometry. All esds (except the esd in the dihedral angle between two l.s. planes) are estimated using the full covariance matrix. The cell esds are taken into account individually in the estimation of esds in distances, angles and torsion angles; correlations between esds in cell parameters are only used when they are defined by crystal symmetry. An approximate (isotropic) treatment of cell esds is used for estimating esds involving l.s. planes.
Refinement. Refinement of *F*^2^ against ALL reflections. The weighted *R*-factor *wR* and goodness of fit *S* are based on *F*^2^, conventional *R*-factors *R* are based on *F*, with *F* set to zero for negative *F*^2^. The threshold expression of *F*^2^ > σ(*F*^2^) is used only for calculating *R*-factors(gt) *etc*. and is not relevant to the choice of reflections for refinement. *R*-factors based on *F*^2^ are statistically about twice as large as those based on *F*, and *R*- factors based on ALL data will be even larger.

### Fractional atomic coordinates and isotropic or equivalent isotropic displacement parameters (Å^2^)

**Table:**

	*x*	*y*	*z*	*U*_iso_*/*U*_eq_	
C1	0.4177 (3)	0.4890 (3)	0.63912 (15)	0.0776 (7)	
H1	0.3408	0.4925	0.5999	0.093*	
C2	0.4854 (3)	0.5845 (2)	0.6769 (2)	0.0889 (10)	
H2	0.4622	0.6629	0.6673	0.107*	
C3	0.5963 (3)	0.5420 (3)	0.7327 (2)	0.0943 (9)	
H3	0.6584	0.5869	0.7668	0.113*	
C4	0.5945 (3)	0.4190 (3)	0.72679 (18)	0.0851 (8)	
H4	0.6566	0.3679	0.7561	0.102*	
C5	0.4835 (3)	0.3867 (2)	0.66940 (16)	0.0754 (7)	
H5	0.4582	0.3106	0.6542	0.091*	
C6	0.2005 (2)	0.39864 (16)	0.80793 (12)	0.0476 (4)	
H6	0.1503	0.3369	0.7822	0.057*	
C7	0.3176 (2)	0.3888 (2)	0.86594 (13)	0.0581 (5)	
H7	0.3582	0.3188	0.8854	0.070*	
C8	0.3639 (2)	0.5015 (2)	0.88996 (12)	0.0623 (5)	
H8	0.4399	0.5188	0.9279	0.075*	
C9	0.2754 (2)	0.58371 (17)	0.84677 (12)	0.0496 (4)	
H9	0.2831	0.6648	0.8510	0.060*	
C10	0.17163 (19)	0.52116 (16)	0.79522 (10)	0.0414 (4)	
C11	0.06791 (18)	0.57653 (15)	0.73633 (12)	0.0437 (4)	
C12	−0.00329 (19)	0.50151 (17)	0.67329 (12)	0.0499 (4)	
H12	0.0185	0.4217	0.6729	0.060*	
C13	−0.0981 (2)	0.54437 (16)	0.61665 (11)	0.0456 (4)	
H13	−0.1167	0.6246	0.6188	0.055*	
C14	−0.17574 (19)	0.47877 (17)	0.55157 (10)	0.0442 (4)	
C15	−0.1691 (3)	0.35727 (18)	0.54580 (13)	0.0583 (5)	
H15	−0.1111	0.3156	0.5838	0.070*	
C16	−0.2464 (3)	0.2974 (2)	0.48517 (15)	0.0749 (7)	
H16	−0.2408	0.2160	0.4829	0.090*	
C17	−0.3305 (3)	0.3563 (2)	0.42873 (15)	0.0774 (7)	
H17	−0.3814	0.3152	0.3874	0.093*	
C18	−0.3413 (3)	0.4771 (2)	0.43210 (14)	0.0698 (6)	
H18	−0.3999	0.5173	0.3936	0.084*	
C19	−0.2647 (2)	0.53742 (19)	0.49291 (12)	0.0556 (5)	
C20	−0.3610 (4)	0.7215 (2)	0.44401 (18)	0.1102 (12)	
H20A	−0.3270	0.7087	0.3882	0.165*	
H20B	−0.3554	0.8036	0.4568	0.165*	
H20C	−0.4638	0.6953	0.4493	0.165*	
O1	0.04308 (17)	0.68202 (11)	0.74047 (9)	0.0631 (4)	
O2	−0.2676 (2)	0.65768 (14)	0.49974 (10)	0.0782 (5)	
Fe1	0.38821 (3)	0.48092 (2)	0.764458 (16)	0.04441 (8)	

### Atomic displacement parameters (Å^2^)

**Table:**

	*U*^11^	*U*^22^	*U*^33^	*U*^12^	*U*^13^	*U*^23^
C1	0.0711 (15)	0.106 (2)	0.0554 (13)	0.0059 (15)	0.0217 (11)	0.0055 (13)
C2	0.095 (2)	0.0698 (16)	0.102 (2)	−0.0105 (15)	0.055 (2)	0.0035 (15)
C3	0.0524 (14)	0.127 (3)	0.103 (2)	−0.0362 (15)	0.0215 (18)	−0.0322 (19)
C4	0.0488 (13)	0.115 (2)	0.0920 (19)	0.0237 (13)	0.0192 (17)	−0.0047 (16)
C5	0.0735 (17)	0.0777 (16)	0.0751 (17)	0.0027 (13)	0.0224 (15)	−0.0200 (13)
C6	0.0461 (11)	0.0452 (9)	0.0514 (11)	−0.0029 (8)	0.0027 (9)	0.0070 (8)
C7	0.0579 (12)	0.0656 (12)	0.0507 (12)	0.0123 (10)	−0.0015 (10)	0.0139 (10)
C8	0.0543 (12)	0.0874 (16)	0.0454 (10)	0.0093 (11)	−0.0110 (9)	−0.0073 (10)
C9	0.0504 (11)	0.0512 (10)	0.0473 (10)	0.0025 (9)	0.0012 (9)	−0.0115 (8)
C10	0.0353 (8)	0.0479 (9)	0.0409 (8)	−0.0001 (8)	0.0049 (7)	0.0008 (8)
C11	0.0371 (9)	0.0465 (9)	0.0474 (10)	0.0036 (6)	0.0046 (8)	0.0028 (8)
C12	0.0429 (10)	0.0527 (11)	0.0540 (10)	0.0055 (8)	−0.0043 (8)	0.0021 (8)
C13	0.0378 (9)	0.0516 (10)	0.0475 (10)	0.0007 (8)	0.0034 (8)	0.0074 (7)
C14	0.0371 (8)	0.0533 (9)	0.0423 (9)	−0.0015 (8)	0.0048 (7)	0.0031 (8)
C15	0.0647 (13)	0.0588 (12)	0.0514 (12)	−0.0030 (10)	0.0029 (10)	0.0065 (9)
C16	0.101 (2)	0.0594 (13)	0.0644 (15)	−0.0176 (12)	0.0059 (15)	−0.0008 (11)
C17	0.0956 (19)	0.0832 (17)	0.0535 (13)	−0.0280 (14)	−0.0101 (14)	−0.0068 (12)
C18	0.0681 (13)	0.0886 (16)	0.0525 (12)	−0.0038 (13)	−0.0143 (10)	0.0043 (12)
C19	0.0510 (11)	0.0675 (13)	0.0484 (11)	0.0012 (10)	−0.0038 (9)	0.0011 (9)
C20	0.152 (3)	0.0934 (19)	0.0852 (19)	0.040 (2)	−0.044 (2)	0.0080 (15)
O1	0.0719 (9)	0.0497 (7)	0.0677 (9)	0.0134 (6)	−0.0116 (8)	0.0000 (7)
O2	0.1015 (14)	0.0633 (9)	0.0698 (10)	0.0214 (9)	−0.0350 (10)	−0.0010 (7)
Fe1	0.03514 (12)	0.04845 (13)	0.04965 (14)	−0.00074 (10)	0.00249 (11)	−0.00507 (10)

### Geometric parameters (Å, º)

**Table:**

C1—C2	1.385 (4)	C9—Fe1	2.0326 (18)
C1—C5	1.391 (3)	C9—H9	0.9300
C1—Fe1	2.041 (2)	C10—C11	1.463 (3)
C1—H1	0.9300	C10—Fe1	2.0294 (17)
C2—C3	1.416 (4)	C11—O1	1.225 (2)
C2—Fe1	2.032 (3)	C11—C12	1.470 (3)
C2—H2	0.9300	C12—C13	1.332 (2)
C3—C4	1.406 (4)	C12—H12	0.9300
C3—Fe1	2.032 (2)	C13—C14	1.460 (3)
C3—H3	0.9300	C13—H13	0.9300
C4—C5	1.398 (3)	C14—C15	1.390 (3)
C4—Fe1	2.047 (2)	C14—C19	1.400 (3)
C4—H4	0.9300	C15—C16	1.374 (3)
C5—Fe1	2.053 (2)	C15—H15	0.9300
C5—H5	0.9300	C16—C17	1.354 (3)
C6—C7	1.399 (3)	C16—H16	0.9300
C6—C10	1.435 (3)	C17—C18	1.382 (3)
C6—Fe1	2.0306 (18)	C17—H17	0.9300
C6—H6	0.9300	C18—C19	1.376 (3)
C7—C8	1.404 (3)	C18—H18	0.9300
C7—Fe1	2.043 (2)	C19—O2	1.376 (3)
C7—H7	0.9300	C20—O2	1.421 (3)
C8—C9	1.406 (3)	C20—H20A	0.9600
C8—Fe1	2.0496 (19)	C20—H20B	0.9600
C8—H8	0.9300	C20—H20C	0.9600
C9—C10	1.429 (3)		
			
C2—C1—C5	109.0 (2)	C15—C14—C13	122.62 (17)
C2—C1—Fe1	69.79 (15)	C19—C14—C13	120.33 (17)
C5—C1—Fe1	70.60 (14)	C16—C15—C14	121.5 (2)
C2—C1—H1	125.5	C16—C15—H15	119.3
C5—C1—H1	125.5	C14—C15—H15	119.3
Fe1—C1—H1	125.7	C17—C16—C15	120.3 (2)
C1—C2—C3	108.0 (2)	C17—C16—H16	119.8
C1—C2—Fe1	70.46 (14)	C15—C16—H16	119.8
C3—C2—Fe1	69.58 (15)	C16—C17—C18	120.4 (2)
C1—C2—H2	126.0	C16—C17—H17	119.8
C3—C2—H2	126.0	C18—C17—H17	119.8
Fe1—C2—H2	125.5	C19—C18—C17	119.5 (2)
C4—C3—C2	106.9 (2)	C19—C18—H18	120.3
C4—C3—Fe1	70.41 (14)	C17—C18—H18	120.3
C2—C3—Fe1	69.63 (13)	O2—C19—C18	123.08 (19)
C4—C3—H3	126.6	O2—C19—C14	115.64 (17)
C2—C3—H3	126.6	C18—C19—C14	121.3 (2)
Fe1—C3—H3	125.0	O2—C20—H20A	109.5
C5—C4—C3	108.4 (3)	O2—C20—H20B	109.5
C5—C4—Fe1	70.30 (13)	H20A—C20—H20B	109.5
C3—C4—Fe1	69.26 (14)	O2—C20—H20C	109.5
C5—C4—H4	125.8	H20A—C20—H20C	109.5
C3—C4—H4	125.8	H20B—C20—H20C	109.5
Fe1—C4—H4	126.2	C19—O2—C20	118.09 (19)
C1—C5—C4	107.7 (3)	C10—Fe1—C6	41.40 (7)
C1—C5—Fe1	69.67 (13)	C10—Fe1—C3	146.85 (11)
C4—C5—Fe1	69.82 (13)	C6—Fe1—C3	169.96 (12)
C1—C5—H5	126.1	C10—Fe1—C2	115.91 (10)
C4—C5—H5	126.1	C6—Fe1—C2	148.66 (12)
Fe1—C5—H5	126.0	C3—Fe1—C2	40.79 (11)
C7—C6—C10	107.77 (18)	C10—Fe1—C9	41.18 (7)
C7—C6—Fe1	70.39 (12)	C6—Fe1—C9	68.92 (8)
C10—C6—Fe1	69.26 (10)	C3—Fe1—C9	114.23 (10)
C7—C6—H6	126.1	C2—Fe1—C9	109.02 (10)
C10—C6—H6	126.1	C10—Fe1—C1	110.65 (9)
Fe1—C6—H6	125.8	C6—Fe1—C1	117.87 (10)
C6—C7—C8	109.06 (18)	C3—Fe1—C1	67.62 (11)
C6—C7—Fe1	69.43 (11)	C2—Fe1—C1	39.76 (11)
C8—C7—Fe1	70.19 (12)	C9—Fe1—C1	133.16 (10)
C6—C7—H7	125.5	C10—Fe1—C7	68.43 (8)
C8—C7—H7	125.5	C6—Fe1—C7	40.18 (8)
Fe1—C7—H7	126.5	C3—Fe1—C7	130.91 (13)
C7—C8—C9	108.18 (17)	C2—Fe1—C7	170.14 (12)
C7—C8—Fe1	69.69 (12)	C9—Fe1—C7	67.87 (8)
C9—C8—Fe1	69.21 (11)	C1—Fe1—C7	148.88 (10)
C7—C8—H8	125.9	C10—Fe1—C4	171.98 (10)
C9—C8—H8	125.9	C6—Fe1—C4	132.11 (11)
Fe1—C8—H8	126.8	C3—Fe1—C4	40.33 (12)
C8—C9—C10	108.20 (17)	C2—Fe1—C4	67.53 (12)
C8—C9—Fe1	70.51 (11)	C9—Fe1—C4	146.05 (11)
C10—C9—Fe1	69.29 (10)	C1—Fe1—C4	66.89 (11)
C8—C9—H9	125.9	C7—Fe1—C4	109.43 (10)
C10—C9—H9	125.9	C10—Fe1—C8	68.51 (7)
Fe1—C9—H9	125.9	C6—Fe1—C8	68.04 (8)
C9—C10—C6	106.80 (16)	C3—Fe1—C8	107.73 (11)
C9—C10—C11	124.36 (17)	C2—Fe1—C8	131.63 (11)
C6—C10—C11	128.62 (17)	C9—Fe1—C8	40.28 (8)
C9—C10—Fe1	69.53 (10)	C1—Fe1—C8	170.76 (11)
C6—C10—Fe1	69.35 (11)	C7—Fe1—C8	40.11 (9)
C11—C10—Fe1	121.96 (12)	C4—Fe1—C8	115.22 (10)
O1—C11—C10	120.04 (18)	C10—Fe1—C5	133.45 (9)
O1—C11—C12	122.19 (17)	C6—Fe1—C5	110.56 (10)
C10—C11—C12	117.77 (15)	C3—Fe1—C5	67.68 (11)
C13—C12—C11	121.99 (18)	C2—Fe1—C5	67.16 (10)
C13—C12—H12	119.0	C9—Fe1—C5	172.29 (10)
C11—C12—H12	119.0	C1—Fe1—C5	39.73 (10)
C12—C13—C14	126.90 (18)	C7—Fe1—C5	117.05 (11)
C12—C13—H13	116.6	C4—Fe1—C5	39.88 (10)
C14—C13—H13	116.6	C8—Fe1—C5	147.23 (10)
C15—C14—C19	117.04 (18)		

### Hydrogen-bond geometry (Å, º)

**Table:**

*D*—H···*A*	*D*—H	H···*A*	*D*···*A*	*D*—H···*A*
C6—H6···O1^i^	0.93	2.48	3.368 (2)	159

Symmetry code: (i) −*x*, *y*−1/2, −*z*+3/2.
